# Traffic Patrolling Routing Problem with Drones in an Urban Road System

**DOI:** 10.3390/s19235164

**Published:** 2019-11-25

**Authors:** He Luo, Peng Zhang, Jiajie Wang, Guoqiang Wang, Fanhe Meng

**Affiliations:** 1 School of Management, Hefei University of Technology, Hefei 230009, China; 2 Key Laboratory of Process Optimization and Intelligent Decision-Making, Ministry of Education, Hefei 230009, China; 3 Engineering Research Center for Intelligent Decision-making & Information Systems Technologies, Ministry of Education, Hefei 230009, China; 4 Key Laboratory of Urban ITS Technology Optimization and Integration, The Ministry of Public Security of China, Hefei 230088, China; 5 Anhui Keli Information Industry CO., LTD, Hefei 230088, China

**Keywords:** traffic patrolling, vehicle and drone coordination, task assignment, joint path planning

## Abstract

The remarkable development of various sensor equipment and communication technologies has stimulated many application platforms of automation. A drone is a sensing platform with strong environmental adaptability and expandability, which is widely used in aerial photography, transmission line inspection, remote sensing mapping, auxiliary communication, traffic patrolling, and other fields. A drone is an effective supplement to the current patrolling business in road traffic patrolling with complex urban buildings and road conditions and a limited ground perspective. However, the limited endurance of patrol drones can be directly solved by vehicles that cooperate with drones on patrolling missions. In this paper, we first proposed and studied the traffic patrolling routing problem with drones (TPRP-D) in an urban road system. Considering road network equations and the heterogeneity of patrolling tasks in the actual patrolling process, we modeled the problem as a double-layer arc routing problem (DL-ARP). Based on graph theory and related research work, we present the mixed integer linear programming formulations and two-stage heuristic solution approaches to solve practical-sized problems. Through analysis of numerical experiments, the solution method proposed in this paper can quickly provide an optimal path planning scheme for different test sets, which can save 9%–16% of time compared with traditional vehicle patrol. At the same time, we analyze several relevant parameters of the patrol process to determine the effect of coordinated traffic patrol. Finally, a case study was completed to verify the practicability of the algorithm.

## 1. Introduction

With the rapid growth of urban vehicle ownership [1], road traffic congestion has become a hotly debated public concern, and traffic management departments can effectively alleviate traffic congestion by deploying patrol vehicles to patrol different road sections [2]. However, when roads are heavily congested, it is difficult for patrol vehicles to enter the congested roads to disperse traffic [3]. In recent years, as a new type of flight platform, drones [4,5] have been used, with the characteristics of being low cost and having a low weight, high mobility and great adaptability to carrying different types of loads [6]. Drones can cooperate with ground vehicles to complete multiple traffic patrolling tasks [7,8,9] and have gradually become effective vehicles for traffic patrolling tasks [10]. For example, both the Seattle and Los Angeles police departments have made drone patrols part of their law enforcement activities; in 2016, the Nanjing Yangtze River road administration brigade manually operated drones to conduct road patrolling and traffic monitoring tasks during holidays, and in 2017, the Devon, Cornwall, and Dorset police forces jointly established the UK’s first dedicated drone force to provide corresponding police services. Therefore, the patrol for traffic monitoring [11] and incident response can use drones to enhance efficiency [12]. However, most of the drones available for patrolling tasks are rotary-wing drones, which have a limited endurance. A direct solution is to use a vehicle to provide assistance [13]. The vehicle transports the drone and performs traffic patrols through the release and recovery of the drone, while the vehicle also performs mission visits. This method takes full advantage not only of the long-distance driving ability of vehicles but also of the high mobility and wireless remote-control aspects of drones [14]. However, this also creates a new problem, which is how to cooperate between a drone and a vehicle during the patrol tasks. Moreover, the collaborative access strategy and route selection of vehicles and drones is a complex optimization problem, and there are still many challenges in the path planning of vehicles and drones.

Presently, most of the current research work focuses on the single point of mission access in collaboration with vehicles and drones, which is often described as a variant of the Traveling Salesman Problem (TSP) [15,16,17]. Murray and Chu et al. [15] first studied the one-vehicle-one-drone coordination problem. The researchers proposed the Flying Sidekick Traveling Salesman Problem (FSTSP), which is a variant of the TSP targeted at different points, but their research [18,19,20,21] is not suitable for the one-vehicle-one-drone coordination problem of traffic patrols. First, although drones are not bound to roads [22,23], for security purposes and road patrolling requirements, drones also need to be under the equations of road networks; thus, as with vehicles, drones also need to have independent and complete flight paths, and the determination of take-off and landing points will clearly not only be a task point but will also be present in any road patrol vehicle path node. Second, the traffic patrolling task is essentially determined by the road network, which is heterogeneous. Finally, due to the occurrence of line tasks in traffic patrolling tasks, the distance between tasks is no longer a fixed value (this phenomenon will be explained in detail in Section 4.1). These specific factors make the joint design of task assignment and path planning quite challenging.

Based on the above analysis, this paper studies the traffic patrolling routing problem with drones (TPRP-D) in an urban road system, which is oriented toward heterogeneous tasks. The road network is no longer just a connection between tasks, it is also the task itself. In this scenario, there are multiple task points and task lines that need to be patrolled. The patrolling task is cooperatively completed by a patrol vehicle and a drone that can take off and land on the patrol vehicle [24]. It takes time for the drone to take off and land. As the drone takes off and lands, the vehicle needs to stay and wait for the drone to complete its operation. The batteries were replaced when the drone was recycled. So the drone has full endurance on every flight. The purpose is to minimize the total mission visit time by optimizing the vehicle’s driving path, the drone’s take-off and landing positions and the drone’s flight path. The main innovation points of this paper are as follows.

(1) A novel problem, the TPRP-D in an urban road system, is introduced and defined. This problem considers the access to heterogeneous tasks in the coordination of vehicles and drones. The problem also considers the road influence on the path. With time minimization as the objective, this process considers all heterogeneous tasks and returns to the patrol center.
(2) This paper models this problem as a double-layer arc routing problem (DL-ARP). By introducing a directed graph as a modeling form of the road network, heterogeneous tasks and two types of vehicle paths in the road network are expressed uniformly. Based on this approach, the patrolling process is modeled and described through two-layer mixed integer programming (MIP).
(3) In this paper, a two-stage heuristic algorithm is designed to solve the TPRP-D. Numerical experiments demonstrate the performance of the algorithm. Finally, a case study is completed on the road network in Hefei, Anhui Province. The research results show that the algorithm designed in this paper can obtain a high-quality feasible solution of the TPRP-D in a relatively short time for the actual-scale problem and quickly provide a satisfactory vehicle–aircraft cooperative traffic patrolling scheme for the traffic police department.

The rest of this article is organized as follows: the existing research is described in Section 2. In Section 3, we illustrate our problem in detail with a mathematical model of mixed integer linear programming. In Section 4, we describe the proposed two-stage heuristic algorithm in detail. In Section 5, we provide a numerical example to demonstrate the effectiveness of the model and the algorithm. In Section 6, we examine a specific application case. In Section 7, conclusions are drawn, and future research directions are discussed.

## 2. Related Work

In recent years, traffic patrol optimization has attracted the attention of many scholars. Adler et al. [25] studied the patrol vehicle center location method based on accident probability hotspots to improve the quick response ability of an entire road network. Lou et al. [26] examined a highway service patrol deployment plan to alleviate congestion. Li et al. [27] also studied the use of a discrete time simulation to evaluate the cost-benefit ratio in areas with a low traffic flux for highway patrol and safety rescue optimization. In the field of transportation, many scholars focus on the prediction, evaluation [28], and prevention of traffic events. They paid relatively little attention to the research of task assignment and path planning for specific patrol tasks. With the expansion of urban scale, a large number of heterogeneous patrol tasks have been generated, and path planning for road patrol tasks is a challenging research direction.

A vehicle is a kind of traditional patrol platform. Under the condition that the current patrol capability is far from enough, a drone is an effective supplement. A drone is a new type of air patrol platform, which can observe the traffic situation on the road with a larger perspective from the air, and can also reach the accident scene or the congested road section quickly, with strong flexibility. There are also a number of studies on the application of drones [29] in traffic patrol optimization. Oh et al. [30] used a drone to examine the road network search problem in the Dubins path case when a group of unconnected roads need to be searched. Chow et al. [22] proposed the deterministic multiperiodic arc inventory routing problem and deployed a drone for traffic monitoring in view of the monitoring requirements of traffic big data. Campbell et al. [31] established the arc routing problem of drones to solve the application of side targets in the access network. When drone capacity is limited, multiple drones should be considered for joint execution. In traffic patrolling, it is not practical to only use drones to execute all patrolling missions. It is necessary to consider the restrictions of complex urban construction facilities, no-fly zones, drone endurance levels, etc. [32]. Traffic patrols through the cooperation between vehicles and drones is a relatively new problem.

With the widespread application of the new generation of information technology, drone technology has been widely applied [33]. The collaboration between vehicles and drones is a growing concern for researchers [34]. Murray and Chu et al. [15] suggested the FSTSP to solve the collaborative delivery of vehicles and drones. Through path planning of vehicles and drones, the mission objectives are completely considered. A two-stage heuristic solution framework is proposed that can effectively obtain the optimal programming results. Agatz et al. [35] were among the first to propose the TSP-D (Traveling Salesman Problem with Drone). The researchers modeled the problem as an integer programming problem and suggested several fast paths by heuristic algorithms based on a local search and dynamic programming, thereby verifying the optimization effect of drone collaboration on the entire process. The authors will extend their model in order to take into account multiple trucks (or drones) in the future work. Boysen et al. [36] studied the scheduling problem of launching drones from transport vehicles to perform point tasks on a given transport vehicle route and introduced two MIP models and a simple heuristic solution framework to solve this problem. Due to the road network, it is more difficult to model the take-off and landing points of drones, which means the solution space of this new problem will be huge. This is the key difference between our work and others. Es Yurek et al. [37] provide an approach for solving the TSP-D and highlight that they are able to solve instances with 10–12 vertices in shorter computation time. Ha et al. [38] considered a variant of the FSTSP that aims to minimize operational costs. These include variable costs for driving and flying, and may include the time spent waiting for trucks and drones. Notably, they also tested their heuristics under the minimal time goal. According to their experiments, under the lowest cost target, the cost was reduced by about 30% (and the maximum completion time increased by about 50%) by truck alone. In addition, under the minimum completion time target, the maximum completion time was reduced by approximately 10% (costs typically reduced by 20%). Ulmer et al. [39] considered factors such as the speed limit of vehicles in cities, geographical partitioning, heterogeneous vehicles, drone performance, and time windows, modeled these elements as a Markov decision-making process, and established a solution method based on geographical regions to increase the total delivery volume in a day and to reduce the total cost of task completion. Luo et al. [40] studied the two-level path problem of a ground vehicle and a drone. The drone can access targets outside the roads, while the vehicle needs to drive within the road network and can intersect the drone path at any time. A new 0-1 integer programming model is established, which considers the routing equations and the interaction between the two levels of routing, and two heuristic algorithms are proposed. Based on Luo’s work, Hu et al. [41] studied the cooperation approach between one vehicle and several drones to complete the path optimization problem of off-road mission target access and proposed an algorithm based on the vehicle-assisted multi-unmanned aerial vehicle (VAMU) algorithm to solve the problem.

Most of these studies start from the actual demands of the logistics distribution and solve the problem of the vehicle–drone cooperative TSP. However, in the traffic patrolling problem, the road not only connects the tasks but also is a task itself; therefore, it is difficult to ignore the influence of the road network on problem modeling and algorithm design. After considering the influence of the road network, the modeling method of heterogeneous tasks and drone tracks will be vastly different, which is the main modeling and algorithm design innovation in this paper.

Table 1 shows the research status of a representative part of relevant work. Obviously, the research on vehicle–drone collaboration is mainly aimed at point task at the beginning, which is a variant of TSP. The study of Arc Route Problem has been extended from CPP (Chinese Postman Problem) and RPP (Rural Postman Problem). Most of these studies are based on ground vehicles. In recent years, there have been a lot of researches on drone Arc Routing Problem. However, there are few researches on vehicle–drone collaboration in road network. Much of the research on the collaboration between vehicles and drones is to use vehicles to move in the road network and drones to visit the target points outside the road network. The problem studied in this paper obviously depends on the road network. But the difference is that the heterogeneous tasks we focus on are the points and lines within the road network.

## 3. Mathematical Modeling

The problem described in this article is the following: the vehicle carries a drone, which can take off and land on the vehicle many times and leaves from the patrol center. Multiple heterogeneous tasks along the road network are accessed. When the vehicle and drone perform joint patrols, the vehicle releases the drone at certain road junctions, and then the vehicle will retrieve the drone at certain junctions. There are time costs associated with both the release and recovery events. Both the vehicle and the drone return to the patrol center. Patrolling missions have been fully considered in this process in both serial and parallel ways. This section provides relevant variable definitions about urban road networks and heterogeneous tasks. At the same time, the TPRP-D is described in detail, and a mathematical model is established.

### 3.1. Description of the Urban Road Network

**Figure 1:**
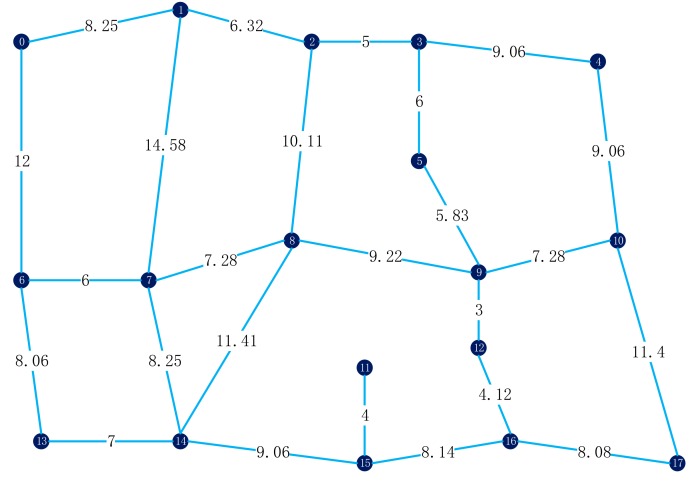
Urban road network.

The discrete road network is simplified as a connected graph, $G=\left(V,E\right)$. Clearly, the set of road intersections is the point set $V=\left(V_{0},V_{1},\ldots ,V_{v-1}\right)$ of connected graph G, where v is the number of points. The set of road sections is the edge set $E=\left\{e_{ij}=\left(V_{i},V_{j}\right)\right\}$ of connected graph G, and the number of edges is  e. Each side $e_{ij}\in E$ has a nonnegative weight, $w\left(e_{ij}\right)$, which represents the length of the edge. If $e_{ij}\notin E$, then $w\left(e_{ij}\right)=0$. Moreover, based on the actual road conditions and convenience of the subsequent modeling, the connected graph is designed as a directed graph, i.e., $e_{ij}\neq e_{ji}$.In this paper, we abstract complex traffic hubs such as overpasses and three-level roads into nodes in the directed graph, and establish a simplified directed diagram of urban road network, as shown in Figure 1. The overpasses do not affect the form of connection between the roads. It does not affect the path decision, which is why we chose to simplify it. However, the existence of urban overpasses affects the vehicle speed, which is reflected in the average vehicle passage speed.

### 3.2. Description of the Heterogeneous Task

In the above directed graph of the road network, we define $V_{0}$ as the patrol center, which serves as the start and end points of the patrol. The point task is expressed as $\;T^{V}=\left(T_{1}^{V},T_{2}^{V},\ldots ,T_{m}^{V}\right)$, for $T^{V}\subseteq V$. In addition to $T_{k}^{V}=V_{i}$, $k=\left\{1,2,\ldots ,m\right\}$, for $i=\left\{1,2,\ldots .v\right\}$, and m≤v−1. Similarly, the line task is expressed as $T^{E}=\left(T_{1}^{E},T_{2}^{E},\ldots ,T_{n}^{E}\right)$, for $T^{E}\subseteq E$, and $T_{k}^{E}=e_{ij}=\left(V_{i},V_{j}\right)$, for $k=\left\{1,2,\ldots ,n\right\}$ and $\left(V_{i},V_{j}\right)\in E$. In addition, n≤e.

### 3.3. Mathematical Formulation

First, we assume that both the vehicles and drones are traveling at a uniform speed in the urban road network and that the time consumed per unit distance is expressed in terms of $C_{V}$ and $C_{U}$, respectively. For a certain city size, the patrol vehicles can be considered to have an unlimited endurance. The maximum range of the drone is expressed as $R_{Max}$. It will also take time to release and recover the drones. We use $S_{L}$ to represent the time that it takes for the drone to be released and $S_{R}$ to represent the time that it takes for the drone to be recovered. Since both the vehicles and drones need to leave and eventually return to the patrol center during the entire process, $V_{0}$ is visited twice. Therefore, we set the point where all vehicles and drones start from as $V_{S}=\left\{V_{0},V_{1},\ldots ,V_{v-1}\right\}$ and the point where all vehicles and drones return to as $V_{E}=\left\{V_{0},V_{1},\ldots ,V_{v}\right\}$. Both $V_{0}$ and $V_{v}$ represent the patrol center. The vehicle path constraints, drone path constraints, patrol time constraints, and task access constraints are described in detail in the remaining four parts of this section.

#### 3.3.1. Vehicle Path Equations

Let the 0-1 variable, $x_{ij}=\left\{0,1\right\}$, indicate whether the vehicle travels from i to j, and $i\in V_{S}$, $j\in V_{E}$, and $\left(V_{i},V_{j}\right)\in E$. In the process of task execution, the following constraints should be satisfied for the vehicle:

(1) $$\underset{\left(i,k\right)\in E}{\sum }x_{ik}=\underset{\left(k,j\right)\in E}{\sum }x_{kj},\forall V_{k}\in V$$

(2) $$\underset{\left(0,j\right)\in E}{\sum }x_{0j}=1,\forall V_{j}\in V_{E}$$

(3) $$\underset{\left(i,0\right)\in E}{\sum }x_{i0}=1,\forall V_{i}\in V_{S}$$

Equation (1) ensures that the inward arc of all nodes in the network is equal to the outward arc, and equation (2) ensures that the vehicle only leaves once from the patrol center, while equation (3) ensures that the vehicle returns to the patrol center only once. This is the fundamental equation for closed loops in graph theory. For any point k in a loop, there is one and only one incoming line segment, which is the outgoing line segment.

The vehicle is the take-off and landing platform of the drone. The vehicle path point set (namely, the set of drone take-off and landing points) is a set of road network points that varies according to the different vehicle paths. Therefore, we define the following auxiliary decision variables to help us model the problem. First, we define the point set of the vehicle’s access path as P, for P⊆V. Second, we set $u_{i}$, whose value represents the order in which the vehicles are accessed to $0\leq u_{i}\leq v+2$, for i∈P. For example, if the path of the vehicle is 0→3→5→1→0, then $u_{0}=1$, $u_{3}=2$, $u_{5}=3$, and $u_{1}=4$. If road network node i is not accessed by vehicles, then $u_{i}=0$, for $i\in \left(V-P\right)$. Equation (4) guarantees that $u_{i}$ increases along the sequence of vehicle access, and equation (5) guarantees that the starting point is always $T_{0}$.

(4) $$u_{i}-u_{j}+1\leq \left(v+1\right)\left(1-x_{ij}\right),\forall V_{i}\in V_{S},V_{j}\in \left\{V_{E}:j\neq i\right\}$$

(5) $$u_{0}=1$$

#### 3.3.2. Drone Path Equations

Based on the auxiliary decision variables set in Section 3.3.1, we set $F_{ij}^{f}=\left\{0,1\right\}$ depending on whether the f sortie of the drone takes off from i and lands in j, $i\in V_{S}$, $j\in V_{E}$, and  f∈N. We define the set of points through which the drone takes off and lands as Q, for Q⊆P. Let $y_{ij}^{f}=\left\{0,1\right\}$ be the f flight of the drone, from i to j, for $\left(V_{i},V_{j}\right)\in E$,f∈N. For the access path of the drone, there are the following constraints:

(6) $$u_{j}>u_{i},\forall F_{ij}^{f}$$

(7) $$\left(u_{j}^{f^{\prime }}-u_{i}^{f}\right)\left(u_{i}^{f^{\prime }}-u_{j}^{f}\right)>0,\forall F_{ij}^{f^{\prime }}$$

(8) $$\left(u_{i}^{f^{\prime }}-u_{i}^{f}\right)\left(f^{\prime }-f\right)>0,\forall F_{ij}^{f^{\prime }}$$

(9) $$\underset{\left(i,k\right)\in E}{\sum }y_{ik}^{f}=1,\forall F_{ij}^{f}$$

(10) $$\underset{\left(k,j\right)\in E}{\sum }y_{kj}^{f}=1,\forall F_{ij}^{f}$$

(11) $$\underset{\left(i,k\right)\in E}{\sum }y_{ik}^{f}=\underset{\left(k,j\right)\in E}{\sum }y_{kj}^{f},\forall V_{k}\in V$$

Equation (6) means that the vehicle must execute a release operation for each sortie before retrieving the drone, and equation (7) guarantees the independence between sorties; that is, the drone must be recovered by the vehicle before any subsequent release. Equation (8) restricts the sequence of sorties to increase along the vehicle direction, and equations (9) and (10) ensure that each drone flight has one departure and one return. Equation (11) ensures that each drone has a unique flight route.

#### 3.3.3. Patrol Time Equations

In Section 3.3.1 and Section 3.3.2, we defined the vehicle and drone path equations in the road network directed graph. Since our goal is to minimize the total time required to complete mission visits and return to the patrol center, calculating the patrol time is an essential step. Based on the vehicle path, the total time consumption is calculated when it passes through each path point. Since the drone takes off and lands at the vehicle’s path point, we can recalibrate the vehicle’s time at these special network nodes. Coordinated traffic patrolling with vehicles and drones is essentially a dynamic combination of the two modes. In one mode, the vehicle is driven in the urban road network, and the drone is located on the vehicle. This mode is called serial patrolling. The other mode is when the drone performs other patrolling tasks alone, while the vehicle is also patrolling. This mode is called parallel patrolling. Therefore, let $t_{i}\;$ be the time that the vehicle arrives at i, for i∈V. Moreover, let $t_{i}^{\prime f}$ be the time when the drone arrives at i in the f  sortie, for i∈V.

(12) $$t_{k}^{\prime f}\geq t_{h}^{\prime f}+w\left(e_{hk}\right)C_{U}-M\left(1-y_{hk}^{f}\right)\;\forall h\in V_{S},\forall k\in \left\{V_{E}:k\neq h\right\},\forall F_{ij}^{f}$$

(13) $$t_{k}^{\prime f}\leq t_{h}^{\prime f}+w\left(e_{hk}\right)C_{U}+M\left(1-y_{hk}^{f}\right)\;\forall h\in V_{S},\forall k\in \left\{V_{E}:k\neq h\right\},\forall F_{ij}^{f}$$

(14) $$t_{i}^{\prime f}=0,\forall F_{ij}^{f}$$

(15) $$t_{k}\;\;\;\;\geq \left\{\begin{array}{c} t_{h}+w\left(e_{hk}\right)C_{V}+S_{L}(\underset{\left(V_{k},V_{j}\right)\in E}{\sum }F_{kj}^{f})+S_{R}(\underset{\left(V_{m},V_{k}\right)\in E}{\sum }F_{mk}^{f^{\prime }})-M\left(1-x_{hk}\right),t_{h}+w\left(e_{hk}\right)C_{V}\geq t_{m}+t_{k}^{\prime f^{\prime }}\\ t_{i}+t_{k}^{\prime f^{\prime }}+S_{L}(\underset{\left(V_{k},V_{j}\right)\in E}{\sum }F_{kj}^{f})+S_{R}(\underset{\left(V_{m},V_{k}\right)\in E}{\sum }F_{mk}^{f^{\prime }})-M\left(1-x_{hk}\right),t_{h}+w\left(e_{hk}\right)C_{V}<t_{m}+t_{k}^{\prime f^{\prime }} \end{array}\right.\;\forall V_{h}\in V_{S},\forall V_{k}\in \left\{V_{E}:k\neq h\right\},\forall F_{kj}^{f},\forall F_{mk}^{f^{\prime }}$$

(16) $$t_{k}\;\;\;\;\leq \left\{\begin{array}{c} t_{h}+w\left(e_{hk}\right)C_{V}+S_{L}(\underset{\left(V_{k},V_{j}\right)\in E}{\sum }F_{kj}^{f})+S_{R}(\underset{\left(V_{m},V_{k}\right)\in E}{\sum }F_{mk}^{f^{\prime }})+M\left(1-x_{hk}\right),t_{h}+w\left(e_{hk}\right)C_{V}\geq t_{m}+t_{k}^{\prime f^{\prime }}\\ t_{i}+t_{k}^{\prime f^{\prime }}+S_{L}(\underset{\left(V_{k},V_{j}\right)\in E}{\sum }F_{kj}^{f})+S_{R}(\underset{\left(V_{m},V_{k}\right)\in E}{\sum }F_{mk}^{f^{\prime }})+M\left(1-x_{hk}\right),t_{h}+w\left(e_{hk}\right)C_{V}<t_{m}+t_{k}^{\prime f^{\prime }} \end{array}\right.\;\forall V_{h}\in V_{S},\forall V_{k}\in \left\{V_{E}:k\neq h\right\},\forall F_{kj}^{f},\forall F_{mk}^{f^{\prime }}$$

(17) $$t_{0}=0$$

(18) $$R_{Max}\geq \left\{\begin{array}{c} \frac{t_{k}^{\prime f}}{C_{U}},t_{h}+w\left(e_{hk}\right)C_{V}\geq t_{i}+t_{k}^{\prime f}\\ \frac{t_{h}+w\left(e_{hk}\right)C_{V}-t_{i}}{C_{U}},t_{h}+w\left(e_{hk}\right)C_{V}<t_{i}+t_{k}^{\prime f} \end{array}\right.\;\forall h\in V_{S},\forall k\in \left\{V_{E}:k\neq h\right\},\forall F_{ij}^{f}$$

First, we calculate the flight time of each drone sortie individually. Equation (14) uniformly sets the time from each take-off point to 0, and equations (12) and (13) ensure that the flight time of the drone in the same sortie increases in sequence according to the flight direction. Second, we calculate the time of the vehicle at each of its path points, and equations (15) and (16) limit the time increase of the vehicle in the entire closed-loop path. At every point in the vehicle path, the status of the drone, whether the drone is at the take-off or landing point, should be judged according to $F_{ij}^{f}$. Then, according to the state of the drone at each vehicle path point, the time of departure from this point and the heading to the next path point is calculated. Equation (17) restricts the starting point time of the vehicles at 0, while equation (18) ensures that the drone flight meets the range limit in each sortie. The equation also considers the situation in which the drone needs to hover and wait in the air for the vehicle.

#### 3.3.4. Task Access Equations

Equation (19) ensures that all line tasks are visited by the vehicle or drone at least once, and equation (20) ensures that all point tasks are visited by the vehicle or drone at least once.

(19) $$x_{ij}+x_{ji}+\underset{f}{\sum }\left(y_{ij}^{f}+y_{ji}^{f}\right)\geq 1,\forall e_{ij}\in T^{E}$$

(20) $$\underset{\left(i,k\right)\in E}{\sum }x_{ik}+\underset{f}{\sum }y_{ik}^{f}\geq 1,\forall V_{k}\in T^{V}$$

### 3.4. Optimized Objective Function of the TPRP-D

To solve the TPRP-D, the optimization problem objective is to minimize the total time that the vehicle and the drone require to jointly perform the tasks.

(21) $$\min t_{v}$$

## 4. Two-Stage Heuristic Algorithm

According to the characteristics of the TPRP-D and the urban road network, in this section, we design a two-stage heuristic algorithm to solve this problem. First, in the first stage of the algorithm, the shortest path of all heterogeneous tasks performed only by the vehicle in the urban road network is solved. By designing a transformation solution method, the problem caused by the uncertain distances between the heterogeneous tasks is solved. Second, in the second stage of the algorithm, based on the shortest path of the vehicle obtained in the first stage, the corresponding task is extracted from the heterogeneous tasks performed by the vehicle and assigned to the drone, and then the path of the vehicle is recalculated. The optimal take-off and landing points of the drone in the vehicle path are calculated when the drone performs the aforementioned task, and the drone’s flight path is designed accordingly. It is determined whether to assign the task to the drone by determining whether this assignment reduces the system time. In this way, optimal task assignment and track planning can be achieved.

Algorithm 1 is an improved two-stage heuristic algorithm. Section 4.1 and Section 4.2 describe the two processes.

**Algorithm 1**. Improved two-stage heuristic algorithm pseudocode.Input: *RoadInfo*, *TaskInfo*;
Output: *VehiRoute, droneRoute*; 
**Begin**
 **//First Stage**
 **OnlyVehiPathFunc**(*RoadInfo*, *TaskInfo*);
 **Obtain**
*VehiRoute*[]; 
 **//Second Stage**
 **HeuristicFunc**(*VehiRoute*[]);
 **Obtain**
*VehiRoute, droneRoute*
**End**

### 4.1. Algorithm Stage 1: Assignment Method for the Heterogeneous Task Distance Matrix

**Figure 2:**
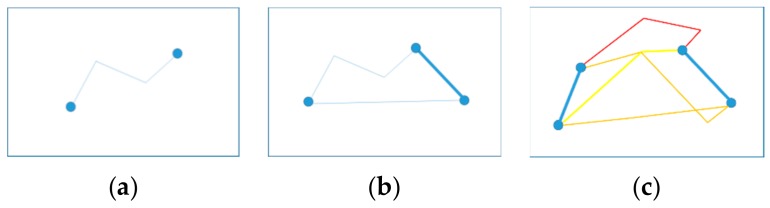
Three ways to connect tasks in the network. (**a**) Point-to-point path, (**b**) Point-to-line path, (**c**) Line-to-line path.

The closed-loop approach to the multiple tasks in urban roads is essentially connecting the tasks in sequence with the lowest cost. Since there are certain differences between point tasks and line tasks, line tasks can be understood as two adjacent point tasks that must be accessed continuously. Patrolling requires entering the road at one of the endpoints and completing the patrol at the other endpoint. Clearly, for heterogeneous tasks in urban road networks, there are three ways to connect tasks in the network (as shown in Figure 2).

**Figure 3:**
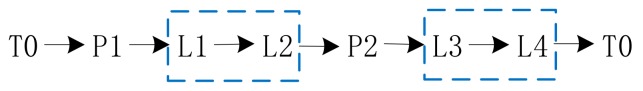
A feasible solution.

For a line task, the difference in direction leads to a different path between the line task and the front-facing and postfacing tasks. In essence, a feasible solution to this problem is the mixed-access order of all heterogeneous tasks (as shown in Figure 3), and the line task is a vector. In this paper, the distance between tasks usually refers to the shortest distance between two points in the urban road network.

If there are N line tasks, then there are $2^{N}$ combinations of fixed-direction line tasks (each line task has two directions). For each task combination, we establish a heterogeneous task distance matrix, where $a_{ij}$ represents the distance from task i to task j in the road network. Clearly, the matrix is asymmetric when line tasks are involved. We present a heterogeneous task distance matrix for the heterogeneous task combination (as shown in Figure 3) in Table 2.

According to Figure 3, we can observe that a task in the feasible solution must have only one pre-task and one post-task. Then, the representation in the heterogeneous task distance matrix is that each row must contain one and only one choice, and so does the column. Their sum is minimized by obtaining the solution to the assignment problem that this matrix represents.

This setting is clearly problematic. Although we set the diagonal elements of the matrix to infinity (INF) (we eliminate the diagonal elements from the solution), the final solution may not be a feasible solution to the original problem. Table 3 shows a case that is a nonfeasible solution to the original problem but a feasible solution to the assigned problem. The number 1 indicates where a solution is obtained.

According to our definition, the two paths expressed in Table 3 are 0→4→3→0 and 1→2→1. Points 1 and 2 form a single closed loop, which means that these points are not accessed. To eliminate this phenomenon, we set up a simple way to translate the unaccessed loop until all tasks can form one loop. This condition means that we need to reduce the values in this matrix, as shown in light green in Table 3. Algorithm 2 is the assignment method for the heterogeneous task distance matrix.

**Algorithm 2.** Pseudocode of the assignment method for the heterogeneous task distance matrix.Input: *RoadInfo, TaskInfo*;
Output: *VehiRoute[]*;
*dIterNum = pow(2, this->iTaskLineNum)*;
**for all**
*dIterNum*
**do**
 *iBinary[iDigits]*=**BinaryConver***(dIterNum)*;
 *Task[TaskNum]*=**Trans***(iBinary[iDigits]);*
  **for all**
*Task[i]*
**do**
  **for all**
*Task[j]*
**do**
   **Dijkstra***(Task[i], Task[j])*;
   **GET**
*dMatrix[i][j]*;
  **end for**
 **end for**
 **while** (true)
  **Hungarian**(*dMatrix[i][j]*);
  **Obtain**
*MinRuting[]*;
  **If (Not Feasible){Modify(***dMatrix[i][j]*)}
  **Else** {break;}
 **end while**
**end for**
**Compare each**
*Minruting[]*;
**Obtain**
*VehiRoute[]*;

The above process involves two solution units used to solve the basic problems. One module is used for calculating the shortest path between two points in the directed graph, namely, the Dijkstra [42] module. The other module is used for solving the assignment problem, namely, the Hungarian [43] module. All of these modules adopt the classic solution method to solve the basic problems.

### 4.2. Algorithm Stage 2: Heuristic Strategy of Drone Task Assignment Optimization

**Figure 4:**
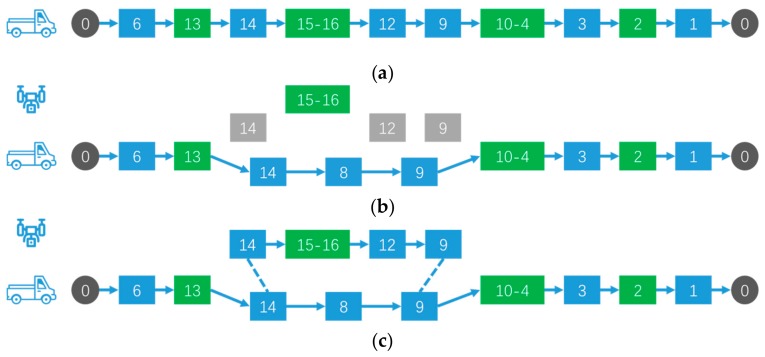
Process of the heuristic strategy of drone task assignment optimization. (**a**): Vehicle traversal path in stage 1, (**b**): Task trial assignment and vehicle path reoptimization, (**c**): Determination of the take-off and landing points and path planning of the drone.

In the first stage, all tasks are assigned to the vehicle, which results in an optimal traversal path. In this section, a heuristic strategy is designed for drone task assignment optimization. Based on the first stage, a complete assignment strategy is finally formed by continuously extracting tasks, redistributing these tasks to the drone and then evaluating the effectiveness. Figure 4 graphically illustrates the heuristic strategy process. The green squares represent task nodes, and the blue squares represent non-task road network nodes.

**Algorithm 3**. Pseudocode for the heuristic strategy of drone task assignment optimization.Input: *VehiRoute*[];
Output: *VehiSubRoute, droneSubRoute*;
**for all** (*task[i]* in *TaskInfo*) **do**
 Call the **CalcVehiSavings**(*task[i]*) function;
 **for all** (*RoutePoint* in *VehiSubRoutes*) **do**
  Call the **CalcdroneStation(***t[i]*,*SubRoute*) function;
  Call the **CalcCost**(*t[i]*,*SubRoute*) function;
 **end for**

 Call the **CalcVehiSavings**(*task[i]*) function;
 **Obtain**
*droneSubRoute*;
 Call the **Update()** function; **end for**
**Obtain**
*VehiSubRoutes*;

First, we traverse all the tasks that have been assigned to the vehicle and assign them to the drone. In each iteration, the time saved by the vehicle not accessing the mission is first calculated. The second step is to calculate the optimal path and landing position for the drone to access this task. If the allocation reduces the total system time, we determine the allocation and the path change. Then, we iterate through the above convenience and trial assignments. Algorithm 3 is the heuristic strategy of the drone task assignment optimization.

## 5. Numerical Experiments

In this section, relevant parameter settings of the subsequent experiments are investigated and determined in Section 5.1 for the research problems mentioned above. In Section 5.2, we identify 9 groups of heterogeneous task settings in the Sioux Falls network and conduct numerical experiments with the designed algorithm to test the performance of the algorithm.

The algorithm was coded with the C++ language. All experiments were conducted in the environment of Microsoft Visual Studio 2015 (Microsoft Corporation, Washington, Redmond, USA) on an x64 Windows 10 desktop computer with a 3.4 GHz i5-7500 CPU and 8 GB of memory.

### 5.1. Parameter Setting

The vehicle performance parameters, drone performance parameters and other relevant parameters are defined in Table 4.

### 5.2. Experimentation

In this section, we develop the solution algorithm described in Section 4 by using Visual Studio. Users can upload road network files and heterogeneous task files to obtain the solution of the problem.

**Figure 5:**
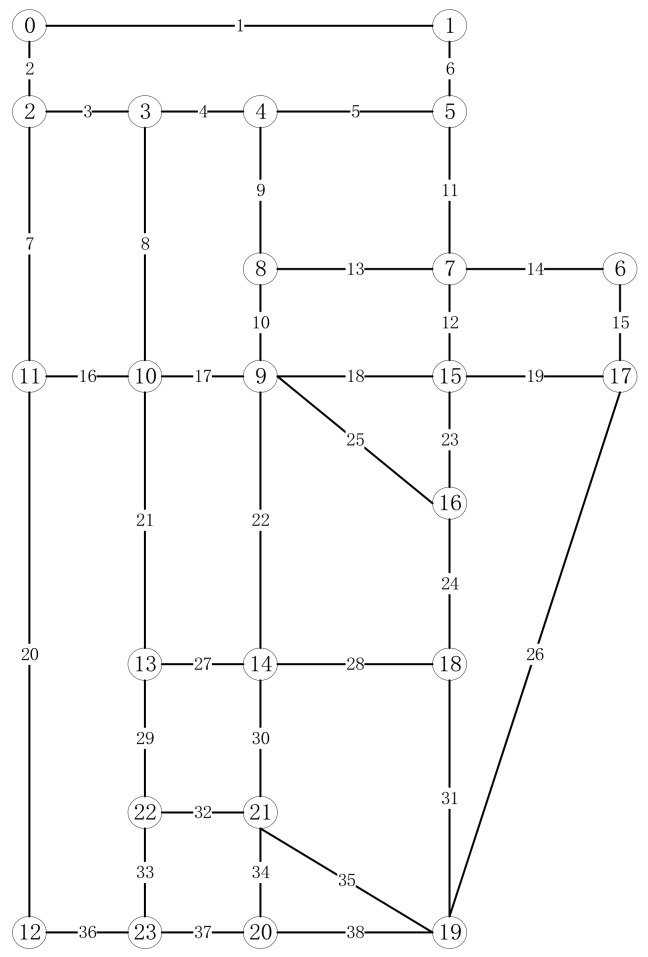
Sioux Falls network [44].

The Sioux Falls network [44] was used for the numerical experiments. The Sioux Falls network consists of 24 nodes and 38 segments, as shown in Figure 5. The length of each segment is listed in Table 5. Points and line segments have separate labels. For example, the length of line 5 is 4, which means that the distance from point 4 to point 5 in Figure 5 is 4. We assume that both the vehicle and the drone are moving at a uniform speed, and we tested various combinations of heterogeneous tasks, including point tasks and line tasks with different numbers and locations.

The test sets of 9 groups of different heterogeneous task settings are summarized in Table 6. The name of the test group indicates the composition of the heterogeneous tasks and the serial number. For example, T23-2 represents that there are two point tasks (point 6 and point 16 in Figure 5) and three line tasks (line 5, line 17, and line 27 in Figure 5).

**Figure 6:**
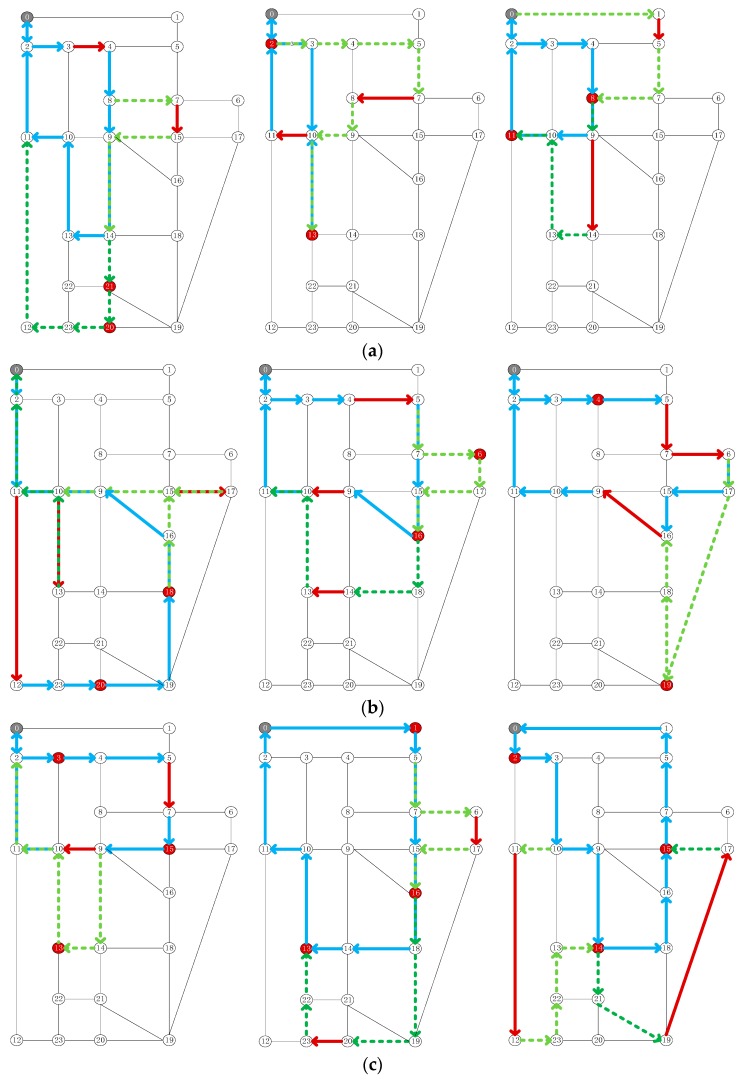
Path planning results. (**a**) T22-1 to T22-1-3, (**b**) T23-1 to T23-3, (**c**) T32-1 to T32-3.

In Table 7, the solution results for the different test sets are given. Taking T23-2 as an example, the final solution result shows that the time of only vehicle obtained is 118.8 min, and the vehicle–drone collaborative time obtained is 100.02 min. The coordinated operation can save 18.78 min. During this vehicle–drone collaborative process, the drone flew two sorts. According to the parameters set in Section 5.1, the preparation time for the take-off and landing of the drone was 24 min, accounting for about 24% of the whole process time. The final path planning results of the 9 groups of test sets are shown in Figure 6. The blue lines indicate the path of the vehicle. The pale green dotted lines represent the path of the drone’s first flight, and the dark green dotted lines represent the path of the drone’s second flight. The gray dots represent patrol centers, the red dots represent point tasks, and the red lines represent line tasks. In T23-2, the vehicle started from point 0, released the drone at point 5, recovered the drone at point 16, and released the drone at the same point after replacing the battery and other operations. The drone returns to the vehicle at point 11 after visiting one mission. Eventually, the vehicle and the drone returned to point 0. During this vehicle–drone collaborative process, the path point of the vehicle is: 0→2→3→4→5→7→15→16→9→10→11→2→0. The path point of the first drone sortie is: 5→7→6→17→15→16. The path point of the second drone sortie is: 16→18→14→13→10→11→2→0.

As shown in Table 7, for the 9 groups of heterogeneous task settings, the joint implementation of patrol tasks by vehicles and drones will reduce the total response time by approximately 12–18 min, and the optimization effect is between 9% and 16%.

It is easy to see that the take-off and retrieval times individually increased according to the timeline. According to the parameter settings in Section 5.1, each flight will have 12 min of drone preparation time. In these 9 test sets, the drone preparation time accounts for approximately 11% to 30% of the total time. If the take-off and landing processes of drones are automated, it will take little or no time. The optimization effect will be increased to between 21% and 42%.

After analysis, the following variables affect the solution of the TPRP-D: vehicle speed; drone speed; drone take-off and landing preparation time; and road network breadth. When the tasks occur in a smaller road network, the vehicle–drone collaboration is irrelevant because the vehicle can quickly access multiple missions and even the entire road network. The advantages of vehicle–drone collaboration only become apparent when the task takes place within a larger road network. At the same time, as the road network gets larger, drone takeoffs and landings become more insignificant, and their impact on total time becomes smaller and eventually leads to the emergence of collaborative efficiency.

## 6. Case Study

**Figure 7:**
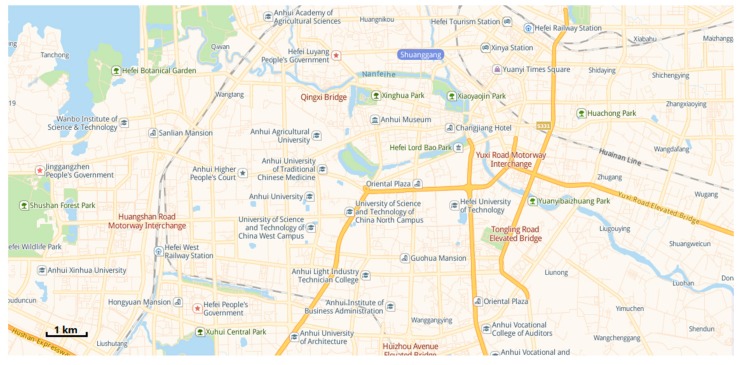
Main road system of Hefei.

**Figure 8:**
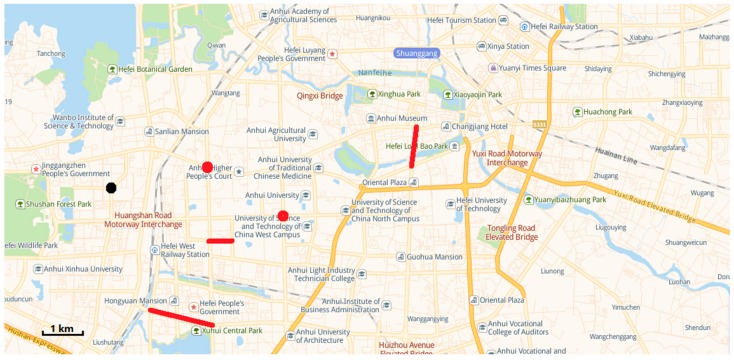
Patrol task setting.

We have worked closely with relevant departments to use existing drones and patrol vehicles to patrol the relevant roads in Hefei, Anhui Province. The urban road traffic area we need to focus on is shown in Figure 7. In this case study, we discussed with relevant departments for many times and finally identified several "hot spots", as shown in Figure 8.

**Figure 9:**
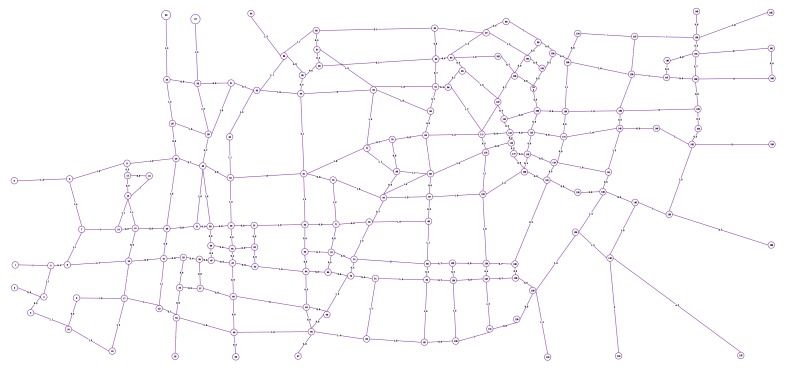
Simplified digital model of the Hefei road network.

**Figure 10:**
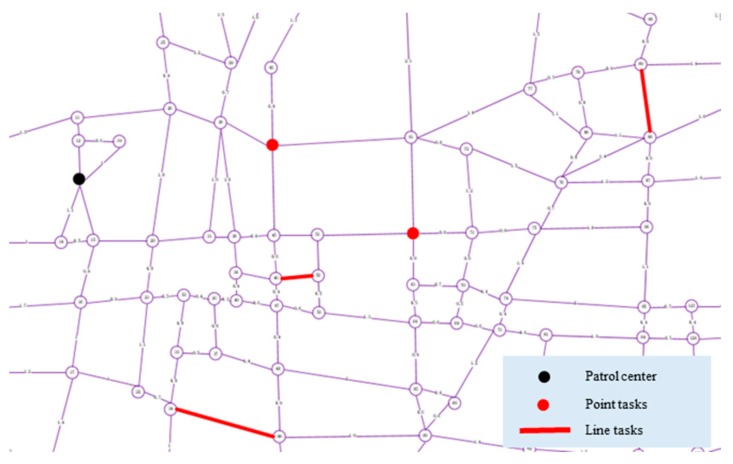
Heterogeneous task setting.

To illustrate the application of the above research work in the real world, a digital model (as shown in Figure 9) of an urban area (as shown in Figure 7) in Hefei, Anhui Province, which includes 163 points and 261 line segments, was built. According to the actual patrolling requirements, heterogeneous tasks are set up, as shown in Figure 10.

**Figure 11:**
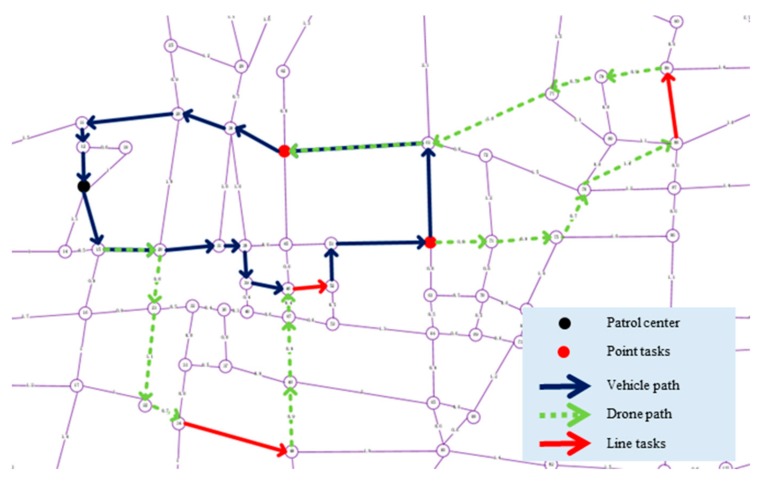
Algorithm solution results.

According to the algorithm tested in Section 5, we use the solution algorithm described in this paper to solve the above task set, and get the results as shown in Table 8. The concrete representation of the solution results in the map is shown in Figure 11.

By analyzing the results of the case study, we found that in the actual application process, the patrol path scheme can be quickly obtained to meet the actual needs by setting the real speed, endurance capacity of the drone, take-off and landing times, and other parameters. It is an effective way to use drone to patrol multiple heterogeneous tasks in the city. Compared with using only vehicle for patrol, the drone makes the whole patrol process more efficient and can effectively reduce the impact of traffic conditions on patrol quality. For the broad urban road network, the vehicle and drone coordinated patrol has a greater advantage.

## 7. Conclusions and Future Work

Based on the reality of road patrolling and the development of drone technology, this paper proposes the TPRP-D in the urban road system, which is modeled as a DL-ARP, and a simple and efficient two-stage algorithm is designed to solve this problem. From the perspective of numerical experiments and case studies, drones can greatly enhance the flexibility and effectiveness of traffic patrolling tasks, and the cooperation between vehicles and drones is the most direct and effective patrolling mode in the current practical work. Traffic patrolling with drones can effectively improve the efficiency of parallel access and reduce the negative impacts of traffic conditions on the patrolling process.

This work lays a foundation for future research on traffic patrolling in collaboration with various vehicles and drones. In this paper, fixed patrolling tasks are considered, while the influences of dynamic time window tasks and the dynamic slave vehicle communication speed on the optimization problem will be interesting research areas. In addition, designing an algorithm for the joint optimization of vehicle and drone routes, such as designing a feasible solution gene operator, is the key to solving large-scale problems. Designing an algorithm for the joint optimization of vehicle and drone routes is still challenging in this field of study.

## Figures and Tables

**Table 1:** Summary of related work.

Reference	Application	Vehicles	Drones	Objective	Task Type	Network	Contribution
Boysen et al. [36]	logistics	1	m	Time	Point	No	MILP, SA
Murray and Chu et al. [15]	logistics	1	1	Time	Point	No	MILP, Heuristic
Agatz et al. [35]	logistics	1	1	Time	Line	Yes	IP, DP, Heuristic
Ulmer et al. [39]	logistics	0	m	Benefit	Line	Yes	MILP, Heuristic
Luo et al. [40]	logistics	1	1	Time	Point	Yes	IP, Heuristics
Hu et al. [41]	logistics	1	m	Time	Point	Yes	IP, VAMU
Es Yurek and Ozmutlu [37]	logistics	1	1	Time	Point	No	Heuristic
Ha et al. [38]	logistics	1	1	Cost/Time	Point	No	MILP, GRASP
This Work	Road Patrol	1	1	Time	Point, Line	Yes	MILP, Heuristic

**Table 2:** Heterogeneous task distance matrix.

	T0	P1	P2	L1-L2	L3-L4
T0	INF	$w\left(e_{T0,P1}\right)$	$w\left(e_{T0,P2}\right)$	$w\left(e_{T0,L1}\right)$	$w\left(e_{T0,L3}\right)$
P1	$w\left(e_{T0,P1}\right)$	INF	$w\left(e_{P1,P2}\right)$	$w\left(e_{P1,L1}\right)$	$w\left(e_{P1,L3}\right)$
P2	$w\left(e_{T0,P2}\right)$	$w\left(e_{P1,P2}\right)$	INF	$w\left(e_{P2,L1}\right)$	$w\left(e_{P2,L3}\right)$
L1-L2	$w\left(e_{T0,L2}\right)$	$w\left(e_{P1,L2}\right)$	$w\left(e_{P2,L2}\right)$	INF	$w\left(e_{L2,L3}\right)$
L3-L4	$w\left(e_{T0,L4}\right)$	$w\left(e_{P1,L4}\right)$	$w\left(e_{P2,L4}\right)$	$w\left(e_{L1,L4}\right)$	INF

**Table 3:** A non-feasible solution.

	T0	P1	P2	L1-L2	L3-L4
T0		↓	↓		1
P1	↓		1	↓	↓
P2	↓	1		↓	↓
L1-L2	1	↓	↓		
L3-L4		↓	↓	1	

**Table 4:** Relevant parameter settings.

Parameters	Brief Description	Value
$C_{V}$	The vehicle speed	30 km/h
$C_{U}$	The drone speed	60 km/h
$S_{L}$	The time it takes to release the drone	6 min
$S_{R}$	The time it takes to recover the drone	6 min
$R_{Max}$	Drone endurance	30 min

**Table 5:** Sioux Falls network.

Segment	Distance	Segment	Distance	Segment	Distance
1	9	14	3.7	27	2.5
2	2	15	2.3	28	4
3	2.5	16	2.5	29	3
4	2.5	17	2.5	30	3
5	4	18	4	31	5.5
6	2	19	3.7	32	2.5
7	6	20	12	33	2.5
8	6	21	6.5	34	2.5
9	3.7	22	6.5	35	4.7
10	2.3	23	3	36	2.5
11	3.7	24	3.5	37	2.5
12	2.3	25	5	38	4
13	4	26	12.5		

**Table 6:** Ten groups of text sets.

Test Group	Point Tasks	Line Tasks
T22-1	20, 21	4, 12
T22-2	2, 13	13, 16
T22-3	8, 11	6, 22
T23-1	18, 20	19, 21, 20
T23-2	6, 16	5, 17, 27
T23-3	4, 19	11, 14, 25
T32-1	3, 13, 15	11, 17
T32-2	1, 13, 16	15, 37
T32-3	2, 14, 15	20, 26

**Table 7:** The solution results.

Test Group	Vehicle Time Consumption (min)	Collaborative Time Consumption (min)	Time Savings (min)	Number of Drone Flights
T22-1	115.98	102	13.98/12.05%	2/23.5%
T22-2	94.02	79.98	13.98/14.87%	1/15.0%
T22-3	94.02	81.72	12.3/13.08%	2/29.4%
T23-1	156.78	138.48	18.3/11.67%	2/17.3%
T23-2	118.8	100.02	18.78/15.81%	2/24.0%
T23-3	120.42	108.6	11.82/9.82%	1/11.0%
T32-1	94.02	82.02	12/12.76%	1/14.6%
T32-2	130.8	118.98	11.82/9.04%	2/20.2%
T32-3	142.8	127.92	14.88/11.6%	2/18.8%

**Table 8:** Single-vehicle patrol test results.

Test Stage	Path Planning Result		Time Cost
Algorithm stage 1	Vehicle path	0 15 20 21 22 34 49 48 47 46 52 51 62 71 75 76 88 89 79 77 61 44 30 26 11 12 0	73.8 min
Algorithm stage 2	Vehicle path	0 15 20 31 38 39 46 52 51 62 51 45 44 30 26 11 12 0	54.84 min
	Drone path 1	15 20 21 22 34 49 48 47 46	
	Drone path 2	62 71 75 76 88 89 79 77 61 44	
